# Injuries in Korean Elite Taekwondo Athletes: A Prospective Study

**DOI:** 10.3390/ijerph17145143

**Published:** 2020-07-16

**Authors:** Boae Son, Young Jae Cho, Hee Seong Jeong, Sae Yong Lee

**Affiliations:** 1 Department of Physical Education, Yonsei University, Seoul 03722, Korea; sonjon11@naver.com; 2 Department of Rehabilitation Medicine, SOL Hospital, Seoul 07592, Korea; cyjjyc94@hanmail.net; 3 International Olympic Committee Research Centre KOREA, Yonsei University, Seoul 03722, Korea; 4 Institute of Convergence Science, Yonsei University, Seoul 03722, Korea

**Keywords:** Taekwondo, epidemiology, athletes, injury surveillance system, martial arts

## Abstract

This prospective cohort study aimed to identify the incidence and characteristics of Taekwondo-related injuries according to age, sex, and event type (i.e., practice or competition) based on a web-based injury surveillance system (ISS) with a follow-up period of 12 months. A total of 285 members of the Korea Taekwondo Association who competed in the 2016 season participated. Injury incidence rates (IRs) were calculated per 1000 athlete-exposures (AEs). Injury rate ratios (RRs) with 95% confidence intervals were calculated and compared regarding age, sex, and event type. During the season, 336 injuries were reported, resulting in a rate of 6.31/1000 AEs. The most common location, type, and mechanism of injury in Taekwondo athletes were the foot/ankle, ligament sprain, and contact with another player, respectively. The overall injury IRs associated with Taekwondo practicing and competition were 4.79/1000 AEs and 24.86/1000 AEs, respectively. The overall injury RR related to both practice and competition was higher in youth athletes than in adult athletes. However, adult athletes were more likely to sustain more severe injuries. Korean elite Taekwondo athletes were commonly exposed to injury, especially youth and female athletes. Therefore, continuation of the ISS suggests the development of interventions for preventing injuries sustained by Taekwondo athletes.

## 1. Introduction

The injury incidence rates (IRs) and time loss in injured Taekwondo athletes were second only to those of football players in the 2008 Beijing Olympic Games [1], the highest in the 2012 London Olympic Games [2], and the fourth highest in the 2016 Rio Olympic Games [3]. The IRs of Taekwondo-related injuries have been ranging from 20.6 to 139.5 per 1000 athlete-exposures (AEs) for elite athletes [4], 79.3 per 1000 AEs for elite athletes during competition [5], and 59.9 per 1000 AEs for amateur Taekwondo athletes [6]. The most common injury location is the foot, followed by the lower leg, knee, and thigh [7].

Over the last decade, important rule changes and the application of new techniques have been implemented in official Taekwondo competitions, including changes in the scoring system, introduction of an electronic protector, and application of an electronic headgear system [8]. Since the 2016 Rio Olympics Games, the valid points in the Taekwondo competition rules are one point for a valid punch to the trunk and awarded for every on “Gam-jeom” given to the opponent contestant, two points for a valid kick to the trunk, three points for a valid turning kick to the trunk and a valid kick to the head, and four points for a valid turning kick to the head [8]. These changes can result in different types and severity of injuries during competition and practice. There is a lack of epidemiological studies investigating the effect of the changes in the rules in terms of Taekwondo-related injuries according to age, sex, and event type (i.e., practice or competition) in Taekwondo.

According to a previous study, no significant difference was observed in injury IRs between youth and adult Greek Taekwondo athletes (odds ratio (OR), 0.77; 95% confidence interval (CI), 0.28–2.14) [9,10]. However, youth male athletes had an increased risk of sustaining a cerebral concussion compared with adult male athletes (OR, 5.72; 95% CI, 0.67–49.14) in the National Championship division [11]. By changing the rules, the level of powerful kicks can be reduced because of changes in the technique of pushing (cut kick), which may minimize the risk of sustaining a serious injury, such as a concussion. It is imperative to understand the characteristics of injuries in youth athletes because sustained injuries in youth athletes may lead to the development of chronic injuries, impairing the athlete’s career. However, epidemiological data on youth elite athlete injuries are limited.

In the 2012 London Olympic Taekwondo Games, male athletes had a two times higher risk of injury than female athletes (rate ratios (RRs), 1.9; 95% CI, 1.1–3.5) [2]. It was reported that male athletes sustained significantly more injuries than their female counterparts (79.9 per 1000 AEs vs. 25.3 per 1000 AEs) and that concussions only occurred in male athletes (6.9 per 1000 AEs) [12]. The overall concussion rate in both male and female athletes is likely to decrease after the introduction of the rule change. In the Australian amateur Taekwondo competition, the proportion of head and neck injuries during practice was lower than that during the competition, but the severity of the injuries was greater during practice than during the competition [12], which may reflect the fact that Taekwondo athletes spend more time practicing and are therefore more exposed to injuries during practice than during competition.

There are more than 5.1 million Taekwondo black belt athletes registered at Kukkiwon, and about 45 Taekwondo competition events hosted by the Korea Taekwondo Association (KTA) are held in the country every year [13]. Given that injury characteristics differ according to age, sex, and event type, epidemiological studies should be conducted to provide data that can inform future prevention of injuries [14]. In this study, we aimed to analyze the incidence, characteristics, and risk factors of Taekwondo-related injuries according to age, sex, and event type.

## 2. Methods

### 2.1. Participants

A total of 285 Korean Taekwondo athletes registered at the Korea Taekwondo Association voluntarily participated in this study (Table 1). All participants provided written informed consent in compliance with the Institutional Review Board at Yonsei University (IRB No. 7001988-201610-BR-273-03). We declare that the investigations were carried out following the rules of the Declaration of Helsinki of 1975, revised in 2013.

### 2.2. Study Procedure

From January to December 2016, the injury surveillance system (ISS) (www.yissem.com/GeneralSurvey) was used to extract epidemiological data for all athletes once every 6 months and to monitor injury events in the athletes. This information was entered into the ISS directly. For data collection, we visited each team to instruct the athletic trainers (AT) about how to record accurate data in the ISS. In addition, we collected injury data from athletes who had ever been treated by AT and/or medical staff supervised by ISS officers for any injury at sports medicine specialists (MD) at designated hospitals. Surveillance of the athletes’ exposures to injury was performed every week by the AT or coaches of each team throughout the season.

### 2.3. ISS Form

The 35-item ISS questionnaire comprised items from both the International Olympic Committee and the United States National Collegiate Athletic Association ISS questionnaires [2,3,15,16]. The questionnaire comprised two sections: (1) general information including nine items on personal information such as age, sex, height, weight, history, and years of experience and (2) event information related to Taekwondo, including injury mechanism, when and where the injury occurred, body location of the injury, type of injury, sports-specific items, and time loss due to the injury. The injury report and AE forms are shown in Supplementary Materials A and B.

Only injuries defined as new were recorded (i.e., pre-existing conditions were not recorded), along with recurring musculoskeletal complaints or other medical conditions that might have occurred during competition and practice during the study season [1]. The diagnoses were made by the sports medicine specialist. AEs were defined as an athlete participating in a team-sanctioned event type (practice and/or competition), in which they are exposed to the risk of a sports injury, regardless of the time associated with participation [15]. Time loss was defined as the time between the original injury and return to play at a level that would allow participation in competition and practice [15,17].

### 2.4. Statistical Analysis

Injury IRs were calculated per 1000 AEs of practice or competition [3]. The injury IRs were defined as IR = ∑ injuries/∑ AEs ×1000. The chi-square test was used to examine associations between age, sex, or event type and injury characteristics (injury site, type, mechanism, and time loss). Rate ratios (RRs) with 95% CI were calculated and used to compare differences between competition and practice [18]. A binary logistic regression model was used to identify the relationship between the odds of sustaining the injury and significant predictors (BMI, sex, age, and years of experience). We used the MedCalc statistical software (version 17.2; MedCalc, Mariakerke, Belgium) and regarded two-tailed *p* values of < 0.05 as statistically significant.

## 3. Results

### 3.1. Overall Incidence of Injury

Among the 285 registered athletes (33.68% females), 336 injuries were reported across 53,082 AEs, resulting in an overall rate of 6.31/1000 AEs. A total of 235 injuries (69.94%) occurred during practice, and 101 injuries (30.06%) occurred during competition. It was estimated that the study year comprised 49,019 practice AEs and 4064 competition AEs. Table 1 shows the characteristics of the athletes and the distribution of injuries during practice vs. competition according to sex and age.

Overall, the most common injury location was the lower extremities (74.11%), followed by the upper extremities (17.87%) and the head and trunk (7.75%). Particularly, the foot and ankle were more prone to injury than other body parts (Table 2), and ligament sprains were the most common type of injury (27.38%) (Table 3). Common injury mechanisms included contact with another player (50.89%), followed by non-contact (19.05%) and overuse injuries (14.88%) (Table 4). Furthermore, the most common activity leading to injury was kicking (63.73%), followed by blocking (14.02%) and stepping (6.51%).

### 3.2. Age-Based Injury Differences

The IRs of practice-associated injuries were higher in adult athletes than in youth athletes, whereas the opposite was observed for competition-associated injuries (Table 1). The overall RRs of injury according to age group were higher among the youth athletes than among the adult athletes (RRs, 1.86; 95% CI, 1.17–2.94; *p* = 0.008). The RRs according to age, sex, AEs, and time loss are shown in Table 5.

**Figure 1:**
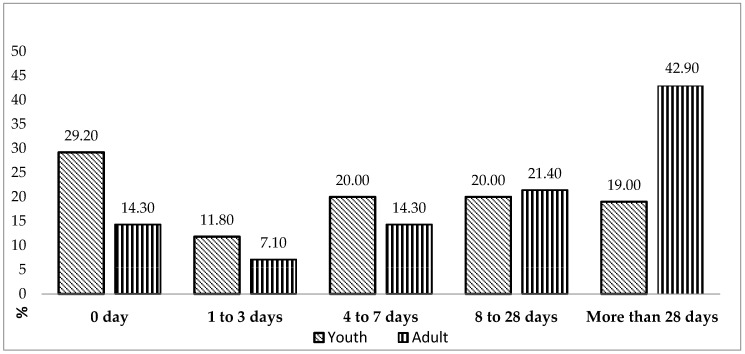
Distribution of time loss due to a Taekwondo-related injury according to age (youth athletes vs. adult athletes).

The extent of injury-associated time loss differed significantly between youth and adult athletes (*χ*^2^ = 12.49; *p* = 0.004; Table 5). The time loss of ≤ 7 days due to injury was also higher in youth athletes, whereas the time loss of >7 days was higher in adult athletes (Figure 1). Adult athletes were 2.82 times more likely to sustain a severe injury than youth athletes (95% CI, 1.66–4.79; Table 5, Figure 1).

### 3.3. Sex-Related Differences in Injury

Regarding the IRs of sex-related injuries according to AEs, IRs were higher in female athletes than in male athletes during practice, whereas the opposite was noted during competition (Table 1). In particular, youth female athletes had higher IRs of injury during practice than their male counterparts. Similarly, the IRs of injury among youth male athletes were substantially higher during competition than those of injury among youth female athletes (Table 1). For adult athletes, differences between male and female athletes were not as pronounced; here, adult female athletes had somewhat higher IRs of injury than adult male athletes both during practice and competition (Table 1). The RRs of injury during practice according to sex were higher in female athletes than in male athletes (RRs, 1.78; 95% CI, 1.08–2.95, *p* = 0.024) (Table 5). No significant difference was observed in injury characteristics between male and female athletes (body site, type, mechanism, and time loss).

### 3.4. Event Type-Based Injury Differences

Regarding event type, the RRs of overall injury were higher during competition than during practice (RRs, 4.97; 95% CI, 3.83–6.40; *p* = 0.001) (Table 5). There was a significant difference between practice and competition in terms of the proportions of injury by a body part (*χ*^2^ = 16.31; *p* = 0.003), type of injury (*χ*^2^ = 26.25; *p* = 0.001), and mechanism of injury (*χ*^2^ = 23.43; *p* = 0.0003). However, there was no significant difference in time loss patterns according to the event type.

### 3.5. Risk Factors for a Taekwondo-Related Injury

None of the factors potentially influencing the risk of sustaining a Taekwondo-related injury exhibited statistically significant between-level differences throughout the year (age, *p* = 0.166; sex, *p* = 0.306; BMI, *p* = 0.964; experience, *p* = 0.741).

## 4. Discussion

This prospective cohort study was conducted for one season using the Taekwondo athletes’ injury surveillance web-based database system with the latest Taekwondo rules integrated, and the results were evaluated according to age, sex, and event type. During the 12-month study period, Korean elite Taekwondo athletes were more likely to sustain an injury of the lower extremities, with ligament sprains being the most commonly encountered type of injury. The most commonly involved exposure to injury was in contact with another athlete. The overall rate ratio for injury was 4.97 times higher during competition than during practice, and the youth athletes had a 1.86 times higher risk of injury than their adult counterparts. Moreover, the female athletes were 1.78 times more likely to sustain an injury during Taekwondo practice than their male counterparts, and youth athletes were 2.00 times more prone to injury than adult athletes during competition. However, severe injuries were more often encountered in adult athletes than in youth athletes.

The overall injury IR per 1000 AEs of Korean elite Taekwondo athletes (6.39; 95% CI, 4.14–5.38) was lower than that observed in Australian amateur Taekwondo athletes (59.93; 95% CI, 51.16–69.77) [6]. The Australian study investigated data on competition-related injuries in amateur athletes over two years, and the present investigation was a prospective study of the data on the practice and competition of elite athletes for one year. In addition to this, the difference observed between the two studies may reflect changes in Taekwondo rules and equipment wearing.

Among the elite Taekwondo athletes in the present study, the most common injury location was the foot, followed by the ankle, knee, and hand/wrist. Moreover, the most common injury type was ligament sprains, followed by contusions, fractures, muscle-tendon strains, and cartilage injuries. In previous studies including elite Taekwondo athletes, data on both the distribution of injuries by site and type of injury exhibited a similar trend [19,20]. Overall, the most common mechanism leading to a Taekwondo-related injury was contact with another player via kicking and blocking. Therefore, the majority of peripheral injuries occurred in the foot, ankle, and hand/wrist, which is to be expected given that these are the common areas of contact when kicking or blocking [6]. Interestingly, compared with the results reported in previous studies, there were no cases of concussions in the present study; the reason for this is unclear but may be attributed to changes in Taekwondo rules and protective equipment [10,19].

Before the new competition rules and protector and scoring system (PSS) were introduced, Taekwondo athletes had a high risk of concussion because of the high score of turning head kicks with very strong power. However, after the Rio summer Olympics Games, electronic body and headgear score systems were introduced to reduce severe injuries, such as concussions and rib fractures [20]. At present, the accuracy of the kick is more important than the force power of the kick for high scores because it is necessary to touch the electronic sensor accurately. Consequently, the strategy now focuses more on using the cut kick technique to achieve a high score [20].

Regarding event type, the total injury IRs in the present study were higher in adult athletes than in their youth counterparts during practice, whereas youth athletes were more prone to injury than their adult counterparts during Taekwondo competitions. Moreover, youth athletes showed higher injury RRs than adult athletes in both practice and competition for a year. The reason for this difference may be associated with the AEs of injury and the proportion of the athletes participating. The trend in injury location, injury type, and mechanism was similar across the age groups; this trend has also been reported previously [6].

Furthermore, the RRs of severe injury were higher among adult athletes than among youth athletes. Similarly, in a previous study, martial arts athletes with more than at least three years of experience were 2.46 times more likely to sustain time loss injuries of ≤ 7 days than those with lesser experience (OR, 2.46; 95% CI, 1.51–4.02) [21]. By changing the rules, the level of powerful kicks may be reduced because of changes in the technique of pushing (cut kicking), which may decrease the risk of severe injuries, such as a fracture and/or concussion, in youth athletes because the force impact of the kick is lower. Therefore, to prevent injuries in youth athletes, it is recommended to plan short term high-intensity interval training and neuromuscular training [22], and to change the age-appropriate Taekwondo policy, such as limiting the time of Taekwondo practice exposure.

The overall injury IRs and/or RRs of injured female athletes were higher than those of their male counterparts during Taekwondo practice. According to Pieter’s study on Taekwondo competition injuries, male athletes were at a higher risk of sustaining competition injuries than their female counterparts (RRs, 1.5; 95% CI, 1.1–2.1; *p* = 0.006) [7]. The reason for this difference is unclear but may be associated with more AEs and the relative weakness of the musculoskeletal system in female athletes. Moreover, this difference may be due to the changes in the Taekwondo rules and PSS. In particular, female athletes are encouraged to prevent ligament sprains and fractures in the lower extremities and wrists/hands and to ensure that their injuries in Taekwondo practice and competition are taken care of.

The strength of the present study was the inclusion of the prospective cohort of elite Taekwondo athletes who completed injury monitoring over a complete one-season study period. The athletes’ response rates in the online surveillance system were very high (98.6% of the total athletes), indicating a strong concern for the topic. We found that the incidence of injury differed significantly according to age and sex. Understanding the characteristics of injuries resulting from training, such as overuse injuries, may suggest preventive actions serving to reduce the incidence of such injuries. Athletes spend far more time training than competing. Therefore, further prospective research should consistently monitor injuries arising not only during competition but also during practice. Koh suggested the continuation of safety education programs on Taekwondo-related concussions for Taekwondo coaches, athletes, referees, and parents to prevent serious injuries in Taekwondo competitions [23].

This study was limited by a potential recall bias because the Taekwondo athletes were asked to consider any injury that they might have suffered over the previous six months during both practice and competition. Moreover, we could not monitor the athletes’ other activities. Therefore, any injuries arising from other activities might have been missed by the athletes and coaches in the report. The issues with the severity of injury were based on estimated time loss rather than the actual time loss. In addition, we recorded only the most severe injury when an athlete presented with multiple injuries; this might have resulted in underestimating the injury incidence and overestimating the severity of injuries. Finally, the findings and conclusions of this study do not allow for generalization since injury monitoring was conducted only for one season.

## 5. Conclusions

Korean elite Taekwondo athletes were frequently exposed to injuries, and youth athletes sustained a higher rate of injuries than adult athletes during practice. However, the proportion of sustained severe injuries was higher in adult athletes than in youth athletes. Moreover, female athletes were more likely to experience an injury than male athletes during practice. Finally, future prospective cohort studies including elite and amateur Taekwondo athletes should rely on data from the ISS and injury prevention programs to provide evidence-based information on risk factors of Taekwondo-related injuries that can be used to adjust the rules for athlete protection.

## Figures and Tables

**Table 1:** Demographics and injury characteristics of Taekwondo athletes participating in the study.

Variables	Youth Athletes			Adult Athletes			Overall		
	Male	Female	Total	Male	Female	Total	Male	Female	Total
No. of athletes	116	67	183	73	29	102	189	96	285
Age (years)	15.20 ± 1.74	15.75 ± 1.62	15.40 ± 1.72	20.22 ± 1.08	19.90 ± 0.99	20.13 ± 1.06	17.15 ± 2.88	17.00 ± 2.40	17.10 ± 2.73
Height (cm)	168.64 ± 10.11	163.76 ± 6.13	166.85 ± 9.17	179.10 ± 5.34	168.14 ± 4.33	175.98 ± 7.08	172.70 ± 9.98	165.09 ± 5.99	170.13 ± 9.54
Weight (kg)	57.43 ± 13.43	53.76 ± 8.86	56.08 ± 12.08	70.93 ± 9.99	60.47 ± 7.81	67.96 ± 10.54	62.67 ± 13.87	55.79 ± 9.09	60.35 ± 12.88
BMI (kg/m^2^)	19.90 ± 3.02	19.93 ± 2.71	19.91 ± 2.91	22.00 ± 2.42	21.30 ± 2.32	21.80 ± 2.41	20.72 ± 2.98	20.34 ± 2.7=68	20.59 ± 2.89
Taekwondo career (years)	2.95 ± 2.26	3.18 ± 2.36	3.04 ± 2.30	8.45 ± 2.68	6.96 ± 2.33	8.03 ± 2.68	5.09 ± 3.62	4.32 ± 2.92	4.83 ± 3.42
No. of total injuries	140	94	234	70	32	102	210	126	336
No. of practice injuries	85	77	162	51	22	73	136	99	235
No. of competition injuries	55	17	72	19	10	29	74	27	101
No. of AEs during practice	21,744	12,563	34,307	10,529	4183	14712	32,509	16,510	49,019
No. of AEs during competition	1655	955	2610	1040	413	1453	2695	1368	4063
Injury IRs/1000 AEs (95% CI), practice	3.91 (3.08–4.74)	6.13 (4.76–7.50)	4.72 (3.99–5.45)	4.84 (3.51–6.17)	5.26 (3.06–7.46)	4.96 (3.82–6.10)	4.18 (3.48–4.89)	6.00 (4.82–7.18)	4.79 (4.18–5.41)
Injury IRs/1000 AEs (95% CI), competition	33.23 (24.45–42.02)	17.80 (9.34–26.26)	27.59 (21.21–33.96)	18.27 (10.05–26.48)	24.21 (9.21–39.22)	19.96 (12.69–27.22)	27.46 (21.20–33.71)	19.74 (12.29–27.18)	24.86 (20.01–29.71)

Note: Values are provided as means ± standard deviations. No.: number, BMI: body mass index, AEs: athlete-exposures, IRs: incidence rates, 95% CI: 95% confidence interval.

**Table 2:** Number of injuries in Taekwondo athletes according to body location.

Body Parts	Youth Athletes	Adult Athletes	Total
Eye	1 (0.43)	0 (0)	1 (0.30)
Nose	1 (0.43)	0 (0)	1 (0.30)
Mouth	1 (0.43)	1 (0.99)	2 (0.60)
Neck	1 (0.43)	1 (0.99)	2 (0.60)
Shoulder	4 (1.70)	1 (0.99)	5 (1.49)
Upper arm	1 (0.43)	0 (0)	1 (0.30)
Elbow	3 (1.28)	1 (0.99)	4 (1.19)
Forearm	0 (0)	2 (1.98)	2 (0.60)
Hand/wrist	38 (16.17)	10 (9.90)	48 (14.29)
Chest	1 (0.43)	2 (1.98)	3 (0.89)
Lower back	13 (5.53)	4 (3.96)	17 (5.06)
Hip/Groin	13 (5.53)	3 (2.97)	16 (4.76)
Thigh	16 (6.81)	6 (5.94)	22 (6.55)
Knee	35 (14.89)	16 (15.84)	51 (15.18)
Shank	11 (4.68)	7 (6.93)	18 (5.36)
Ankle	40 (17.02)	30 (29.70)	70 (20.83)
Foot	55 (23.40)	17 (16.83)	72 (21.43)
Other	1 (0.43)	0 (0)	1 (0.30)
Total	235 (100)	101 (100)	336 (100)

Note: Data are provided as numbers (%).

**Table 3:** Types of injury in Taekwondo athletes.

Variables	Youth Athletes	Adult Athletes	Total
Ligament sprain	54 (22.98)	38 (37.62)	92 (27.38)
Contusion	60 (25.53)	23 (22.77)	83 (24.70)
Fracture/stress fracture	57 (24.26)	14 (13.86)	71 (21.13)
Muscle-tendon strain	18 (7.66)	8 (7.92)	26 (7.74)
Cartilage	11 (4.68)	4 (3.96)	15 (4.46)
Disc	9 (3.83)	3 (2.97)	12 (3.57)
Inflammation	8 (3.40)	2 (1.98)	10 (2.98)
Laceration	1 (0.43)	1 (0.99)	2 (0.60)
Tendinitis	4 (1.70)	2 (1.98)	6 (1.79)
Neurological injury	1 (0.43)	1 (0.99)	2 (0.60)
Dislocation/subluxation	1 (0.43)	2 (1.98)	3 (0.89)
Other	7 (2.98)	1 (0.99)	8 (2.38)
Unknown	4 (1.70)	2 (1.98)	6 (1.79)
Total	235 (100)	101 (100)	336 (100)

Note: Data are provided as numbers (%).

**Table 4:** Mechanisms of injury in Taekwondo athletes.

Variables	Youth Athletes	Adult Athletes	Total
Contact with another players	120 (51.06)	51 (50.50)	171 (50.89)
Non-contact	38 (16.17)	26 (25.74)	64 (19.05)
Overuse	37 (15.74)	13 (12.87)	50 (14.88)
Defense	23 (9.79)	6 (5.94)	29 (8.63)
Falling	11 (4.68)	2 (1.98)	13 (3.87)
Other contact	1 (0.43)	2 (1.98)	3 (0.89)
Unknown	5 (2.13)	1 (0.99)	6 (1.79)
Total	235 (100)	101 (100)	336 (100)

Note: Data are provided as numbers (%).

**Table 5:** Rate ratios according to age, sex, athlete-exposures (AEs), and time loss of Taekwondo athletes.

Variables	Factor	RRs (95% CI)	*p*-Value
Overall	Competition (vs. practice)	4.97 (3.83–6.40)	0.001 *
	Female (vs. male)	1.49 (0.92–2.42)	0.107
	Youth (vs. adult)	1.86 (1.17–2.94)	0.008 *
Practice	Overall female (vs. overall male)	1.78 (1.08–2.95)	0.024 *
	Overall youth (vs. overall adult)	1.82 (1.12–2.96)	0.015 *
	Youth female (vs. youth male)	1.16 (0.89–1.52)	0.258
	Adult female (vs. adult male)	0.95 (0.55–1.64)	0.868
Competition	Overall female (vs. overall male)	0.96 (0.51–1.82)	0.903
	Overall youth (vs. overall adult)	2.00 (1.06–3.60)	0.032 *
	Youth female (vs. youth male)	0.74 (0.41–1.32)	0.315
	Adult female (vs. adult male)	2.23 (0.96–5.23)	0.063
Time loss >7 days	Overall Adult (vs. overall youth)	2.82 (1.66–4.79)	0.001 *
	Overall female (vs. overall male)	1.00 (0.61–1.63)	0.999

Note: RRs (95% CI): risk ratios (95% confidence interval), * *p*-value < 0.05.

## Supplementary Materials

The following are available online at https://www.mdpi.com/1660-4601/17/14/5143/s1: A: Injury Report Form (Taekwondo Athletes) by the Injury Surveillance System IOC Research Centre KOREA and B: Athlete Exposure Form.
