# Supplementary material for: Injuries in Korean Elite Taekwondo Athletes: A Prospective Study
Source: Int J Environ Res Public Health. 2020 Jul 16;17(14):5143. doi: 10.3390/ijerph17145143 (PMC7399793; doi:10.3390/ijerph17145143)
Supplement: Supplementary file 1 [file ijerph-17-05143-s001.pdf]

Appendix A.

**Injury Report Form *(Taekwondo Athletes)* by the Injury Surveillance System**
**IOC Research Centre KOREA**

□ School: ________________

□ Season: (1) March season (1 semester) March-August
(2) Fall Season (2 Semester) September-February

1. Name: __________________

2. Birth (age): __________year _______month _______day

3. Gender: (1) Male (2) Female

4. Your Taekwondo career? (e.g., 8 year 7 months) (___) year (___) month

5. Your menstrual cycle?
(1) Normal menstruation (average menstrual cycle 22–39 days, menstrual period 3–7 days)
(2) Hypomenorrhea (abnormal menstrual cycle for more than 40 days and less than 3 months)
(3) Primary amenorrhea (when menstruation has not started until the age 16)
(4) Secondary amenorrhea (skipping more than three times during practice and/or competition, or no menstruation for more than 6 months)
(99) Others:

6. Height: _________________cm

7. Weight:_________________kg

8. Which side is your dominant side?
- Hand: (1) Right (2) Left (3) Both
- Foot: (4) Right (5) Left (6) Both

9. How many competitions did you participate in for last 6 months?
_________times, _________minute (1 game = 1 time)

10. When was your recent injury? ( _______ month/day)

11. Did you have injuries lately in competition or practice?
(1) Yes (2) No

*(If you chose "Yes" in Question 12, please go to Question 14 and answer to the end, If you answered "No," please proceed as normally)*

12. Over the past 6 months, did your injury occurs in a season or off-season?
(1) In a season

(2) Off-season

13. Were you injured in the competition and/or during practice over the past six months?
(1) Competition
(2) Practice

14. If you were injured recently, when did it occur? Please check all options below that apply.
- Competition: (1) Warm-up (2) 1 Round (3) 2 Round (4) 3 Round (5) Cool-down
- Practice: (6) Dawn (7) Morning (8) Afternoon (9) Evening
(99) Other:

15. For the last 6 months, has your injury been acute or chronic?
(1) Acute (2) Chronic (Overuse)

16. Please choose all body parts where you have been injured or treated in for 6 months.
(1) Eye (2) Nose (3) Mouth (4) Neck (5) Shoulder (6) Upper arm
(7) Elbow (8) Forearm (9) Hand/Wrist (10) Lower back (11) Hip/Groin
(12) Thigh (13) Knee (14) Shank (15) Ankle (16) Foot (17) Other

◎ Concussion Information (Please answer if you checked any of items 1–9 in question 16)

17. What symptoms were diagnosed if you had a concussion

(1) Grade1 Concussion

(No loss of consciousness, memory loss for up to 2 minutes after injury)

(2) Grade2 Concussion

[Unconscious (less than 5 minutes), memory loss for up to 30 minutes after injury]

(3) Grade3 Concussion

[Unconscious (more than 5 minutes), memory loss for a considerable period of time after injury]

(4) No Concussion

(5) Other

18. Did you wear all the protection equipment associated with Taekwondo?

(1) Yes (2) No

19. If you had an eye injury, what was the type of eye injury?

(1) Orbital fracture (2) Corneal damage (3) Eye rupture (4) No eye injury (99) Other:

20. Please choose all types of injuries that you have been diagnosed with by a medical specialist over the past 6 months.

(1) Abrasion (2) Contusion(bruise) (3) Laceration (4) Puncture wound (5) Partial ligament tear (sprain) (6) Ligament complete tear (sprain) (7) Muscle-tendon partial rupture (strain) (8) Muscle-tendon complete rupture (strain) (9) Tendinosis (10) Tendinopathy (11) Muscle spasm (12) Bursitis (13) Synovitis (14) Capsulitis (15) Subluxation (16) Dislocation (17) Cartilage damage (18) Fracture (19) Stress fracture (20) Other bone injury (21) Disc injury (22) Nerve injury (23) Concussion (24) Inflammation (unknown cause) (25) Infection (26) Bleeding (27) Necrosis (28) Blister (29) Boil (30) Hernia (31) Dental injury (32) Corn, Wart (99) Other (Please specify):

21. Has there been any bleeding from the recent injury?

(1) Yes (2) No

22. Was the surgery needed for any recent injuries?

(1) Yes (Season) (2) Yes (Off-season) (3) No

23. If a recent injury occurred during a competition or practice match, did you get injured during attack or defense?

(1) Attack (2) Defense (3) Unknown (4) unrelated to this injury

24. During which particular activity did your recent injury occur? (Please choose all that apply)

(1) Kick (2) Blocking (3) Step (4) Landing (5) Falling (6) Avoidance
(7) Jump (8) Punch (9) Foul (10) Other (11) Unknown

25. What was the mechanism of the recent injury? (Please choose all that apply)

(1) Player-contact (2) Non-contact (3) Overuse (4) Defense (5) Falling
(6) Other contacts (7) Unknown

26. If your recent injury occurred during a competition, please choose a number as close to where you were injured as possible (A is on your team).

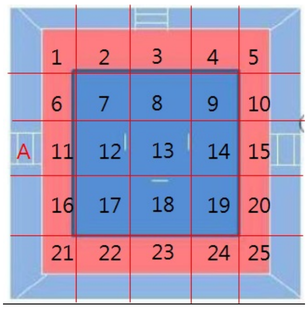


27. How long did it take to recover from the injury and participate in competition or practice?

(1) 0 days (2) 1–3 day(s) (3) 4–7 days (4) 8–28 days (5) More than 28 days

28. How was whether when you had the injury?

(1) Sunny (2) Cloudy (3) Rainy (4) Showers (5) Snowing (6) Thunder and lighting

(7) Foggy (8) Other

29. What type of floor did you use when did you had your recent injury?
(1) Official mat (2) General mat (3) Grass (4) Sand (5) Polyurethane track
(6) Wood (7) Cement (8) asphalt (99) Other:

30. Did your injury affect your performance in your competitions for the next 6 months?
(1) Yes (2) No (3) Do not know

31. Were you interested in treating or preventing the injury?
(1) Very interested (2) Interested (3) Not interested (4) Do not know

32. When did you receive treatment after the injury experienced by you for the past 6 months?
(1) Immediately (2) Within 2–3 days (3) Within 5–6 days (4) Within 10 days (5) Did not receive treatment

33. Please check all the items that apply to the treatment and prevention of the injury experienced by you for the past 6 months.
(1) Medication (2) Physiotherapy (3) Manipulation therapy (4) Rehabilitation therapy
(5) Taping (6) Wearing a protector (7) Massage (8) Cold and hot packs
(9) Oriental medicine (acupuncture etc.) (10) Home remedy
(99) Other (Please specify):

34. Please indicate what you are doing to prevent injury (please check all that apply).
(1) Warm-up (2) Cool-down (3) Regular medical checkup
(4) Personal (rehabilitation) training (5) Nothing special
(99) Other (Please specify):

35. If you do not usually treat or prevent injury, then why is that so?
(1) Tired (2) Busy (3) Don’t know how to do it (4) Cost problems
(5) Don’t feel the need
(99) Other (Please specify):

**Appendix B.**

## Athlete Exposure Form

▣ This is a questionnaire that examines information on injury exposures over a week experienced by the entire team. Please read the questions and select/write down the appropriate answer.

□ The definition of "injury exposure," as defined by the ISS, is when an athlete participates in a training or competition activity

□ NOTE: 1. Please accurately record the number of times you have participated in training or competitions.

2. Please only record information about participation in the official training of the entire team for a week (excluding individual training)

<u>Practice</u>

*<u>Please answer all the questions</u>*

1. Which season is this week?: ___________
   (1) Season
   (2) Off-season
2. The number of training sessions this week: _______________times (assuming once a day from Monday to Sunday)
3. On average, how many did people attend the practice?: _____________________
4. How many times did athletes train at the official taekwondo mat this week?: __________times (assuming once a day from Monday to Sunday)

5 How many times did athletes train at the training ground (i.e., not the mat) this week?: _______________times (assuming once a day from Monday to Sunday)

Competition

7. Was there any game this week? ____________times
   (1) No (Questionnaire ends here)

   (2) Yes (Continue to answer the next question)

8. How many competitions were carried out this week? ______________times (assuming once a competitiont)

| Record how many athletes participated in competition | Location of competition (Check V) | **Stadium floor type** (Check V) |
|---|---|---|
| Competition | | |
| 1.____________(Person) | 1. Home □ Away□ | 1. Official mat □ |
| 2.____________(Person) | 2. Home □ Away□ | 2. General mat □ |
| 3.____________(Person) | 3. Home □ Away□ | 3. No mat □ |
| 4.____________(Person) | 4. Home □ Away□ | |
| 5.____________(Person) | 5. Home □ Away□ | |
